# Inhibition of Coronavirus Entry *In Vitro* and *Ex Vivo* by a Lipid-Conjugated Peptide Derived from the SARS-CoV-2 Spike Glycoprotein HRC Domain

**DOI:** 10.1128/mBio.01935-20

**Published:** 2020-10-20

**Authors:** Victor K. Outlaw, Francesca T. Bovier, Megan C. Mears, Maria N. Cajimat, Yun Zhu, Michelle J. Lin, Amin Addetia, Nicole A. P. Lieberman, Vikas Peddu, Xuping Xie, Pei-Yong Shi, Alexander L. Greninger, Samuel H. Gellman, Dennis A. Bente, Anne Moscona, Matteo Porotto

**Affiliations:** a Department of Chemistry, University of Wisconsin, Madison, Wisconsin, USA; b Department of Pediatrics, Columbia University Medical Center, New York, New York, USA; c Center for Host-Pathogen Interaction, Columbia University Medical Center, New York, New York, USA; d Department of Experimental Medicine, University of Campania “Luigi Vanvitelli,” Caserta, Italy; e Galveston National Laboratory, University of Texas Medical Branch, Galveston, Texas, USA; f Department of Experimental Pathology, University of Texas Medical Branch, Galveston, Texas, USA; g Beijing Pediatric Research Institute, Beijing Children’s Hospital, Capital Medical University, Beijing, China; h Department of Laboratory Medicine, University of Washington School of Medicine, Seattle, Washington, USA; i Department of Biochemistry and Molecular Biology, University of Texas Medical Branch, Galveston, Texas, USA; j Vaccine and Infectious Disease Division, Fred Hutchinson Cancer Research Center, Seattle, Washington, USA; k Department of Microbiology and Immunology, University of Texas Medical Branch, Galveston, Texas, USA; l Department of Microbiology & Immunology, Columbia University Medical Center, New York, New York, USA; m Department of Physiology & Cellular Biophysics, Columbia University Medical Center, New York, New York, USA; St. Jude Children’s Research Hospital

**Keywords:** SARS-CoV-2, spike protein, fusion inhibitor, lipopeptide

## Abstract

The emergence of severe acute respiratory syndrome coronavirus type 2 (SARS-CoV-2), the etiological agent of the 2019 coronavirus disease (COVID-19), has erupted into a global pandemic that has led to tens of millions of infections and hundreds of thousands of deaths worldwide. The development of therapeutics to treat infection or as prophylactics to halt viral transmission and spread is urgently needed. SARS-CoV-2 relies on structural rearrangements within a spike (S) glycoprotein to mediate fusion of the viral and host cell membranes. Here, we describe the development of a lipopeptide that is derived from the C-terminal heptad repeat (HRC) domain of SARS-CoV-2 S that potently inhibits infection by SARS-CoV-2. The lipopeptide inhibits cell-cell fusion mediated by SARS-CoV-2 S and blocks infection by live SARS-CoV-2 in Vero E6 cell monolayers more effectively than previously described lipopeptides. The SARS-CoV-2 lipopeptide exhibits broad-spectrum activity by inhibiting cell-cell fusion mediated by SARS-CoV-1 and Middle East respiratory syndrome coronavirus (MERS-CoV) and blocking infection by live MERS-CoV in cell monolayers. We also show that the SARS-CoV-2 HRC-derived lipopeptide potently blocks the spread of SARS-CoV-2 in human airway epithelial (HAE) cultures, an *ex vivo* model designed to mimic respiratory viral propagation in humans. While viral spread of SARS-CoV-2 infection was widespread in untreated airways, those treated with SARS-CoV-2 HRC lipopeptide showed no detectable evidence of viral spread. These data provide a framework for the development of peptide therapeutics for the treatment of or prophylaxis against SARS-CoV-2 as well as other coronaviruses.

## Abstract (precis)

SARS-CoV-2, the causative agent of COVID-19, continues to spread globally, placing strain on health care systems and resulting in rapidly increasing numbers of cases and mortalities. Despite the growing need for medical intervention, no FDA-approved vaccines are yet available, and treatment has been limited to supportive therapy for the alleviation of symptoms. Entry inhibitors could fill the important role of preventing initial infection and preventing spread. Here, we describe the design, synthesis, and evaluation of a lipopeptide that is derived from the HRC domain of the SARS-CoV-2 S glycoprotein that potently inhibits fusion mediated by SARS-CoV-2 S glycoprotein and blocks infection by live SARS-CoV-2 in both cell monolayers (*in vitro*) and human airway tissues (*ex vivo*). Our results highlight the SARS-CoV-2 HRC-derived lipopeptide as a promising therapeutic candidate for SARS-CoV-2 infections.

## INTRODUCTION

The recently emerged severe acute respiratory syndrome coronavirus 2 (SARS-CoV-2), the causative agent of coronavirus disease 2019 (COVID-19), has infected tens of millions of people leading to hundreds of thousands of deaths worldwide. Despite the growing need for antiviral therapeutics, no FDA-approved treatment or prophylactic measure currently exists.

**FIG 1:**
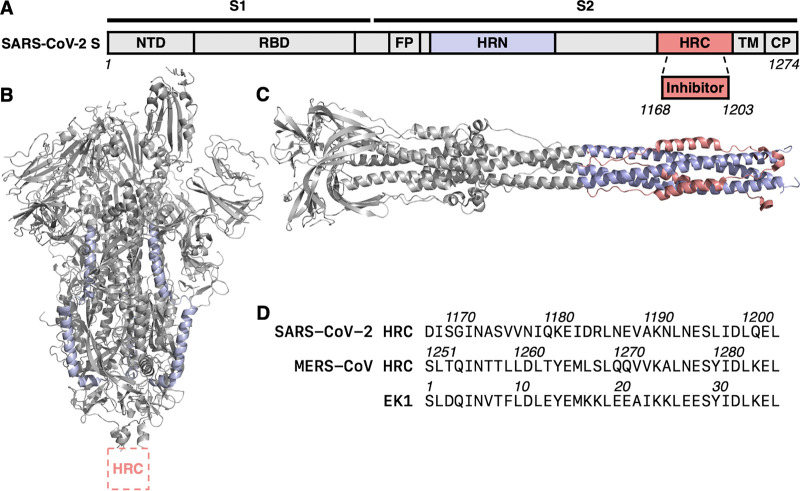
SARS-CoV-2 spike (S) glycoprotein domain architecture and structure. (A) Simplified schematic diagram of SARS-CoV-2 S. The N-terminal domain (NTD), receptor-binding domain (RBD), fusion peptide (FP), N-terminal heptad repeat (HRN), C-terminal heptad repeat (HRC), transmembrane (TM), and cytoplasmic tail (CP) domains are depicted. (B) Prefusion conformation of SARS-CoV-2 S (PDB accession number 6VSB). (C) Model of postfusion conformation of SARS-CoV-2 S based on homology with HCoV-229E S (PDB 6B3O) and SARS-CoV S (PDB 1WYY). (D) Sequences of SARS-CoV-2 HRC, MERS-CoV HRC, and EK1 peptides.

Infection by SARS-CoV-2, as well as other coronaviruses, requires membrane fusion between the viral envelope and cell membrane, a process mediated by the viral envelope spike (S) glycoprotein. Coronaviruses employ a type I fusion mechanism to gain access to the cytoplasm of host cells (1). Other pathogenic viruses that employ the type I fusion mechanism include human immunodeficiency virus (HIV), paramyxoviruses, and pneumoviruses (2–4). SARS-CoV-2 S is a large homotrimer (5, 6). Each monomer consists of two subunits, S1 and S2, which contain several domains (Fig. 1A). A receptor-binding domain (RBD) within the S1 subunit is responsible for cell surface attachment. A cell fusion domain (FD) within the S2 subunit is responsible for mediating membrane merger.

Prior to activation, SARS-CoV-2 S is anchored to the outer viral membrane in a prefusion conformation (Fig. 1B). Upon viral attachment to the target cell (and endosomal uptake in certain cases), host factors, including receptors and proteases, trigger large-scale conformational rearrangements in the FD. The process is initiated by formation of an extended and inherently unstable form of the fusion protein trimer, with the FD becoming inserted into the target cell membrane. The intermediate then rearranges via association of two heptad repeat (HR) domains of the fusion subunit, one near the amino N terminus (HRN) and the other near the C terminus (HRC), into a compact six-helix bundle (6HB) assembly (Fig. 1C). Achieving the stable 6HB assembly drives fusion of the cell membrane and viral envelope that is required for infection (reviewed in references 3, 7, and 8).

Peptides derived from HR domains of viral fusion proteins, including S proteins of several coronaviruses, inhibit infection of the virus from which they were derived. These HR peptides are thought to form 6HB-like assemblies with the intermediate extended form of the fusion protein trimer, thereby disrupting the structural rearrangement of S that drives membrane fusion. We have previously demonstrated that lipid conjugation to HRC-derived inhibitory peptides markedly increases antiviral potency and *in vivo* half-life (9–12). This lipopeptide strategy has been used to create effective entry inhibitors for prophylaxis and/or treatment for human parainfluenza virus type 3, measles virus, and Nipah virus infection in animals (10, 11, 13–16). In addition to enhancing efficacy against viruses that fuse at the target cell membrane, lipid conjugation also enables activity against viruses that do not fuse until they have been taken up via endocytosis (17). For example, potent inhibition of influenza virus infection has been shown by lipopeptides in an animal model (15). In each of these cases, the efficacy in a series of *in vitro* and *ex vivo* assays predicted the concentrations necessary for efficacy *in vivo*. The peptides were nontoxic, retained therapeutic levels in the desired tissues for over 24 h, and were effective when delivered subcutaneously or via direct administration to the airway.

Coronavirus S-mediated fusion can occur either at the cell surface membrane or in the endosomal membrane depending on the cell type and coronavirus strain. Because cholesterol conjugation has been shown to enhance inhibition of fusion both at the plasma membrane and within endosomal compartments, this strategy is particularly attractive for the development of inhibitors of coronavirus fusion. Our previous efforts included development of lipid-conjugated peptides that target Middle East respiratory syndrome coronavirus (MERS-CoV). Park and Gallagher showed that our lipid conjugation design for coronavirus S increases the antiviral potency of MERS-CoV-derived peptides up to 1,000-fold, and the resultant peptides inhibited entry by CoV strains that fuse at the plasma membrane as well as by those that fuse in endosomal compartments (18). Others have also demonstrated that a lipid moiety increased potency for inhibiting S-mediated fusion *in vitro* relative to peptides lacking a lipid (19, 20). These precedents motivated us to determine whether a lipopeptide based on the HRC domain of S from SARS-CoV-2 would inhibit entry by the virus responsible for the current pandemic.

The studies described below compare a lipopeptide containing the SARS-CoV-2 HRC domain with a previously reported MERS-CoV-derived lipopeptide and a lipopeptide derivative of the designed peptide EK1 (Fig. 1D). The EK1 sequence, derived from the human coronavirus HCoV-OC43 HRC domain, has been reported to display inhibition in a SARS-CoV-2 S-mediated cell-cell fusion assay and in a pseudotyped virus infection assay, and has been proposed as a pan-coronavirus inhibitor (21). We find that the SARS-CoV-2 HRC-derived cholesterol conjugate potently inhibits fusion mediated by the SARS-CoV-2 S protein, blocks infection of cultured cells by live SARS-CoV-2 and MERS-CoV, and inhibits the spread of SARS-CoV-2 in human airway epithelia (HAE). The SARS-CoV-2 HRC lipopeptide was more effective than lipopeptides based on either the MERS HRC sequence or EK1 against infectious SARS-CoV-2 and highly effective against infectious MERS as well. The activity displayed by the SARS-CoV-2 lipopeptide is well within the range that has previously correlated with *in vivo* efficacy (10, 16, 22, 23), which suggests that this lipopeptide may offer broad anti-CoV inhibition.

## RESULTS

### Lipopeptide design.

Our inhibitor design focused on a 36-residue peptide corresponding to residues 1168 to 1203 within the HRC domain of the SARS-CoV-2 S protein (Fig. 1D). This segment was chosen based on sequence alignment with a previously described MERS-CoV HRC-derived fusion-inhibitory peptide (18). For lipid conjugation, this 36-residue segment was extended at the C terminus by a Gly-Ser-Gly-Ser-Gly-Cys segment. The cysteine side chain was used as a nucleophilic handle to append a cholesterol unit, with an intervening tetra-ethylene glycol segment. We have previously employed this approach to conjugate fusion-inhibitory peptides to cholesterol and other hydrophobic moieties (10, 11, 13). The cholesterol group is intended to anchor the peptide in cellular membranes. The 42-mer peptide (HRC plus C-terminal extension) was prepared by microwave-assisted solid-phase peptide synthesis protocols, purified by reverse-phase high-performance liquid chromatography (HPLC), conjugated to a bromoacyl tetra-ethylene glycol-cholesterol reagent, as previously described (11, 13), and purified again by HPLC.

Cholesterol-conjugated lipopeptides derived from the MERS-CoV HRC domain (corresponding to residues 1251 to 1286 of MERS-CoV S) and the designed peptide EK1 were prepared using an analogous protocol. EK1, a sequence derived from the HCoV-OC43 HRC domain with modifications to enhance solubility, has been suggested previously as a pan-coronavirus fusion inhibitor (21).

### SARS-CoV-2 HRC lipopeptide is a potent inhibitor of SARS-CoV-2 S-mediated fusion.

Our initial functional evaluation of the SARS-CoV-2 HRC lipopeptide was conducted with a cell-cell fusion assay based on β-galactosidase (β-Gal) complementation that we adapted for assessment of SARS-CoV-2 S-mediated fusion. For this assay, cells expressing human angiotensin-converting enzyme 2 (hACE2) and the N-terminal portion of β-Gal were mixed with cells expressing the SARS-CoV-2 S protein and the C-terminal portion of β-Gal. When fusion mediated by S occurs, the two portions of β-gal combine to generate a catalytically active species, and fusion is detected via the luminescence that results from substrate processing by β-Gal. This assay format allows us to test potential inhibitors of SARS-CoV-2 S-mediated membrane fusion without handling infectious virus.

**FIG 2:**
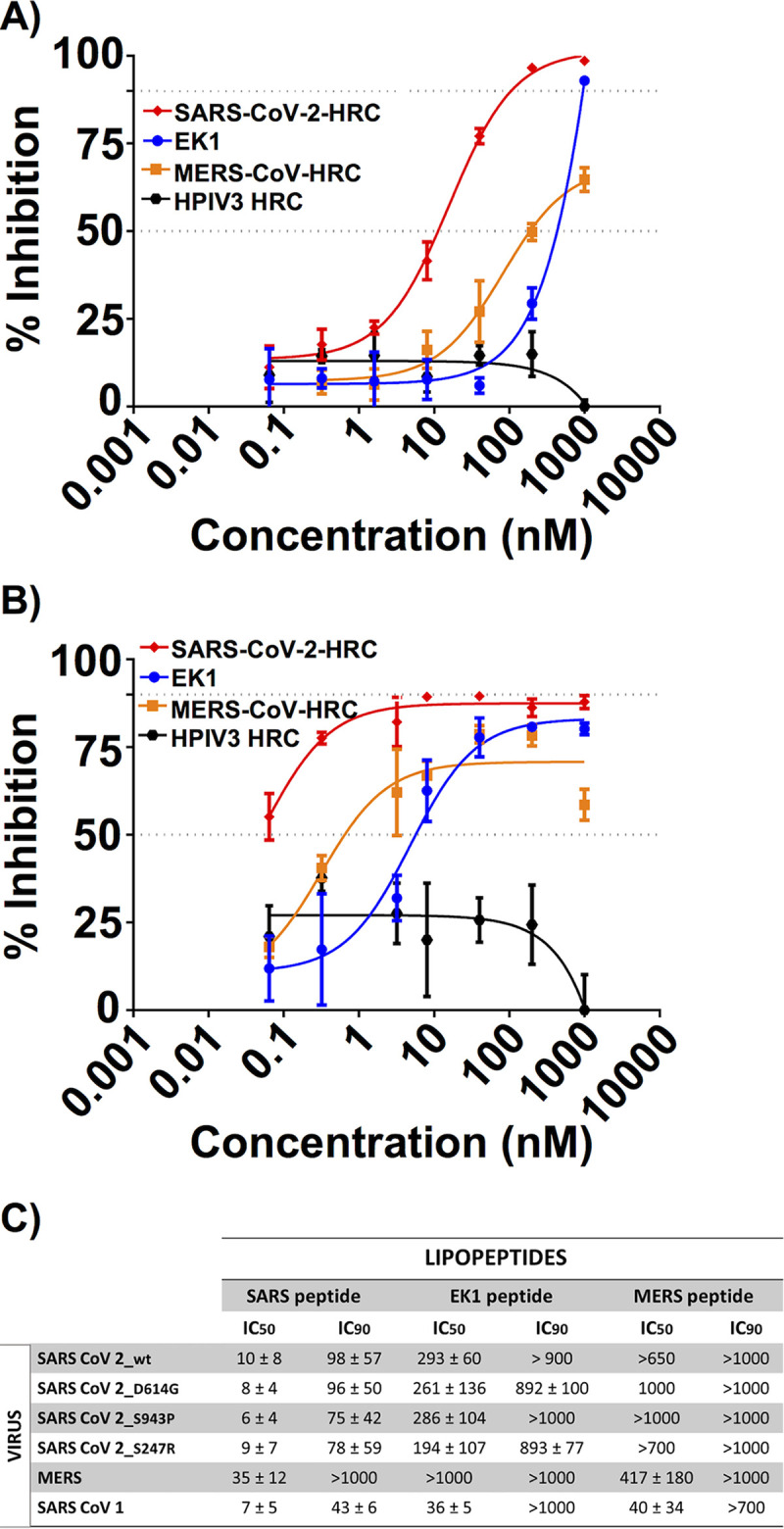
Fusion inhibition assay: inhibiting SARS-CoV-2, SARS-CoV-1, and MERS S-protein-mediated fusion. (A and B) Inhibition of S-mediated fusion in 293T cells with high concentration of ACE-2 receptor (A) or low concentration of ACE-2 receptor (B) by lipopeptides derived from SARS-CoV-2 HRC (red), MERS-CoV HRC (orange), EK1 (blue), or HPIV3 HRC (black). (C) Inhibition of fusion mediated by SARS-CoV-2 S mutants, MERS-CoV S, and SARS-CoV-1 S. Percent inhibition was calculated as the ratio of relative luminescence units in the presence of a specific concentration of inhibitor and the relative luminescence units in the absence of inhibitor and corrected for background luminescence as follows: percent inhibition = 100 × [1 − (luminescence at X − background)/(luminescence in the absence of inhibitor – background)]. Data are means ± standard errors (SE) (error bars) from three separate experiments with the curve representing a four-parameter dose-response model.

Figure 2 shows results from cell-cell fusion assays, where percent inhibition corresponds to the extent of suppression of the luminescence signal that is observed in the absence of any inhibitor (i.e., 0% inhibition corresponds to maximum luminescence signal). The SARS-CoV-2 HRC lipopeptide potently inhibited S-mediated fusion, with 50% inhibitory concentration (IC_50_) of ∼10 nM and 90% inhibitory concentration (IC_90_) of ∼100 nM. A lipopeptide based on the human parainfluenza virus type 3 (HPIV3) F protein HRC domain, used as a negative control, did not inhibit fusion at any concentration tested. The EK1-derived lipopeptide inhibited S-mediated fusion in this assay but was substantially less potent (IC_50_ ∼ 300 nM, and IC_90_ > 900 nM) than the SARS-CoV-2 HRC lipopeptide. The MERS-CoV lipopeptide exhibited only weak inhibition of S-mediated fusion (IC_50_ > 650 nM). These data indicate that the SARS-CoV-2 lipopeptide was 30- to 90-fold more effective than the EK1 lipopeptide at inhibiting fusion mediated by SARS-CoV-2 S, based on IC_50_ and IC_90_ values, respectively.

A 3-(4,5-dimethylthiazol-2-yl)-2,5-diphenyltetrazolium bromide (MTT) assay was performed in parallel during this experiment to evaluate the potential toxicity of each lipopeptide (see Fig. S1A in the supplemental material). Toxicity for each of the lipopeptides was minimal (<20%), even at the highest concentrations tested (5 μM). No toxicity was observed for the SARS-CoV-2 lipopeptide at its IC_90_ concentration (100 nM).

10.1128/mBio.01935-20.2 FIG S1 *In vitro* and *ex vivo* cytoxicity assessment. MTT assay was used to determine the toxicity of the indicated peptides in HEK293T (A), Vero (B), and human airway epithelium (C). Toxicity was evaluated as described in Materials and Methods. Download FIG S1, TIF file, 2.5 MB. Copyright © 2020 Outlaw et al. 2020 Outlaw et al. This content is distributed under the terms of the Creative Commons Attribution 4.0 International license.

Figure 2B shows a fusion assay performed with target human embryonic kidney (HEK) 293T cells that were not transfected with hACE2. These cells, therefore, do not overexpress the viral receptor and provide a model for infection in the presence of a lower level of receptor. With reduced receptor expression, the IC_50_ and IC_90_ values for all the peptides decreased dramatically, suggesting that lipopeptide potency is greater in tissues with lower levels of receptor. However, the potency order among the three lipopeptides did not change in this assay relative to the fusion assay performed with cells overexpressing hACE2.

Despite the overall stability of the SARS-CoV-2 genome, variants with mutations in S have recently spread globally (24–32). These mutations in S resulted in increased infectivity (e.g., D614G [24]) or were located in the putative target domain of the HRC peptide (e.g., S943P). To determine the impact of mutation on efficacy, we examined the ability of our lipopeptides to inhibit fusion mediated by each of these emerging S protein mutants. Figure 2C shows the IC_50_ and IC_90_ values of the three lipopeptides against each of the S mutants. For D614G, which has become predominant in the circulating strain in 2020, as well as the other two S mutants, the SARS-CoV-2 HRC lipopeptide remains substantially more potent than the EK1 or MERS-CoV lipopeptide, without a significant difference compared to the original S sequence.

Finally, we asked whether each lipopeptide displays broad-spectrum activity by assessing their ability to inhibit MERS-CoV and SARS-CoV-1 S-mediated fusion (Fig. 2C). The SARS-CoV-2 HRC lipopeptide was more effective than both MERS-CoV and EK1 lipopeptides at inhibiting fusion mediated by either MERS-CoV or SARS-CoV-1 S.

### SARS-CoV-2 HRC lipopeptide is a potent inhibitor of infectious SARS-CoV-2 and MERS-CoV in monolayer culture.

**FIG 3:**
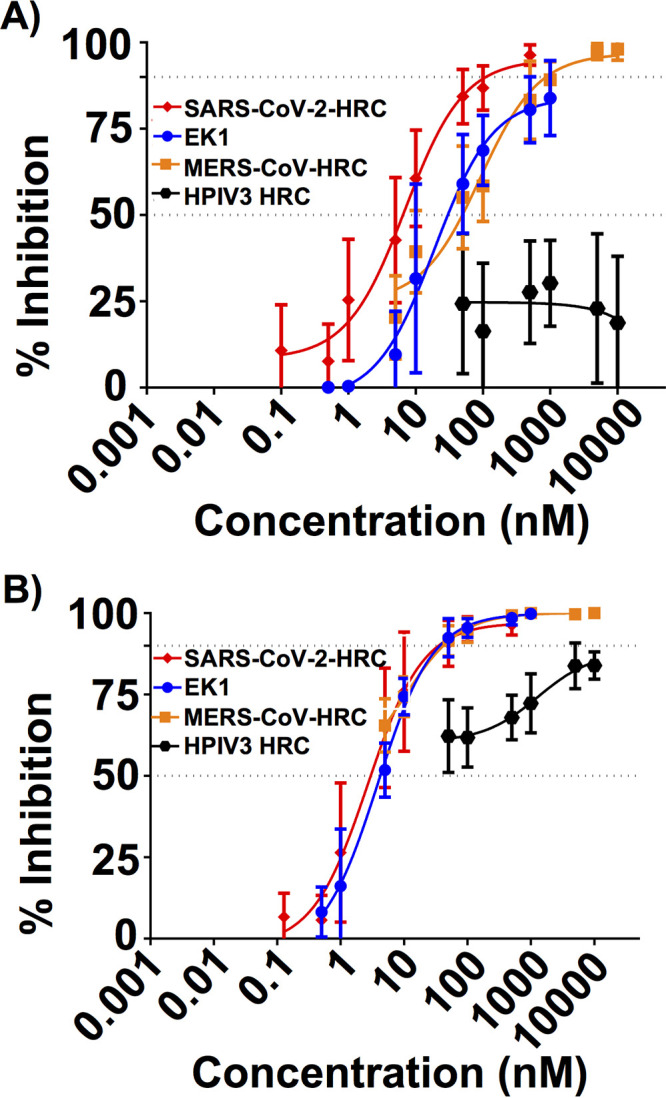
Virus inhibition assay: inhibiting SARS-CoV-2 and MERS-CoV virus infection. (A and B) Inhibition of infection by live SARS-CoV-2 (A) or MERS-CoV (B) by lipopeptides derived from SARS-CoV-2 HRC (red), MERS-CoV HRC (orange), EK1 (blue), or HPIV3 HRC (black). Percent inhibition was calculated as the ratio of PFU in the presence of a specific concentration of inhibitor and the PFU in the absence of inhibitor. Data are means ± SE from three separate experiments with the curve representing a four-parameter dose-response model.

Observation of potent inhibition of S-mediated cell-cell fusion by the SARS-CoV-2 HRC lipopeptide motivated us to ask whether this compound could inhibit SARS-CoV-2 or MERS-CoV infection of cultured cells (Fig. 3). Inhibition was assessed by a plaque neutralization test. In this assay, virus was incubated for 1 h in the presence of lipopeptide at the concentrations shown and then added to monolayers of cultured Vero E6 cells. After 72 h, the plaques were counted to determine the extent of infection. The SARS-CoV-2 HRC lipopeptide was a potent inhibitor of SARS-CoV-2 infection (Fig. 3A; IC_50_ ∼ 6 nM). The HPIV3 F HRC lipopeptide was completely inactive against SARS-CoV-2. The EK1 (IC_50_ ∼ 41 nM) and MERS-CoV (IC_50_ ∼ 36 nM) lipopeptides were substantially less potent than the SARS-CoV-2 HRC lipopeptide against SARS-CoV-2 infection.

The SARS-CoV-2 lipopeptide was also a potent inhibitor of MERS-CoV infection (Fig. 3B; IC_50_ ∼ 3 nM). Both EK1 (IC_50_ ∼ 2 nM) and MERS-CoV (IC_50_ ∼4 nM) lipopeptides were very effective as well at blocking MERS-CoV infection. Interestingly, the HPIV3 lipopeptide also demonstrated activity against MERS-CoV infection although only at higher concentrations relative to the other lipopeptides tested. We have previously demonstrated that HPIV3 HRC-derived peptides can interact with HRN domains and/or block infection by several viruses (10, 11, 13).

Toxicity of lipopeptides in Vero E6 cells was also evaluated by a MTT assay (Fig. S1B). The SARS-CoV-2 (toxicity < 10%) and EK1 (toxicity < 20%) lipopeptides demonstrated minimal toxicity at all concentrations less than 1 μM.

### SARS-CoV-2 HRC peptide inhibits infectious SARS-CoV-2 viral spread in a human airway epithelial *ex vivo* model.

In order to model virus infection in the natural host, we used the human airway epithelium (HAE) model. HAE is ideal for assessing viral spread in experiments that represent the clinical scenario. Unlike monolayer cell cultures, HAE has been shown to recapitulate the selective pressures that influence respiratory viral propagation in humans (33). HAE has been recently used to test small-molecule SARS-CoV-2 inhibitors (34). We have previously shown that HAE is an ideal model to assess fusion-inhibitory peptide activity as well as other antiviral strategies (10, 13, 35–37).

**FIG 4:**
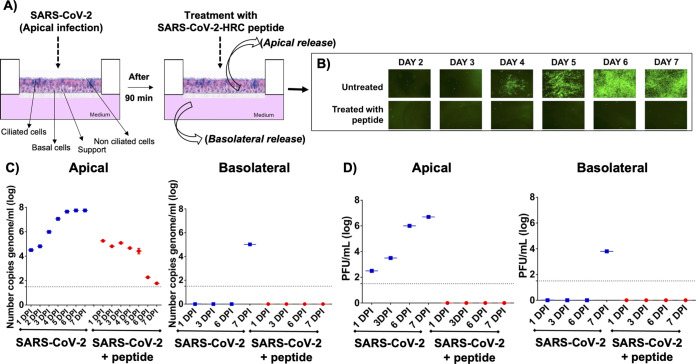
SARS-CoV-2-derived cholesterol-conjugated peptides block SARS-CoV-2-mNeonGreen viral spread in human airway epithelial cells (HAE). (A) HAE cells were infected with SARS-CoV-2 (2,000 PFU/well for a multiplicity of infection of ∼0.02) for 90 min before adding SARS-CoV-2 peptide. Fluid was collected from the apical or basolateral surfaces daily for 7 days (as shown in the schematic in panel A). (B) Spread of fluorescent virus is shown at the indicated days with or without peptide treatment. (C) Viral genome copies in apical or basolateral fluids were determined by RT-qPCR at the indicated time points (days postinfection [DPI]). (D) Infectious viruses released were quantified by titration from the apical or basolateral spaces. The median values are represented by horizontal bars, and the detection limits are indicated by the dotted lines. RT-qPCR and viral titration were performed on supernatant fluids sequentially collected from the same HAE wells the pictures were taken. Data were from three separate wells for infection treated and two separate wells for infection untreated.

To ask whether the SARS-CoV-2 HRC lipopeptide could inhibit the spread of SARS-CoV-2 in human airway tissue, HAE samples in isolated wells were infected with an infectious clone derived from SARS-CoV-2 expressing a stable mNeonGreen reporter gene (icSARS-CoV-2-mNG) (38) for 90 min, a period that permits viral entry (diagrammed schematically in Fig. 4A). Then, SARS-CoV-2 HRC lipopeptide was added to half of the wells (20 μl of 10 μM lipopeptide solution to wells containing 1 ml medium to give a final lipopeptide concentration of 200 nM). Each day, the apical surface was washed with 200 μl of phosphate-buffered saline (PBS), and the supernatant fluid was collected to assess viral titer. Additional lipopeptide aliquots were added after 24 h (day 2) and 48 h (day 3). All samples (treated with lipopeptide and untreated) were monitored by fluorescence microscopy daily through day 7. Representative HAE fluorescence micrographs are shown in Fig. 4B. In the absence of lipopeptide treatment, viral spread became visually evident at day 4, and by day 6, SARS-CoV-2 infection was widespread. In contrast, HAE samples that were treated daily for days 1 to 3 with the SARS-CoV-2 HRC lipopeptide showed no detectable evidence of viral spread during the 7 days. HAE cells incubated for 6 days in the presence of the SARS-CoV-2 HRC lipopeptide at the extremely high concentration of 100 μM showed only ∼20% toxicity (Fig. S1C).

We quantified the virus that spread in the airway tissue each day for 7 days during the time course experiment. Quantitative reverse transcriptase PCR (RT-qPCR) and plaque assays were performed to detect the level of viral genome and the amount of live virus present in the fluid collected from the apical side of the HAE (Fig. 4C and D, left graphs), which corresponds to the air-exposed surface, and from the basolateral side (Fig. 4C and D, right graphs), which represents the circulation-facing surface. Data are shown as the average number of RNA genome copies and average of the plaque-forming units (PFU) per milliliter in supernatant fluid from each HAE well in the presence (red) or absence (blue) of the SARS-CoV-2 HRC lipopeptide. The presence of 10^4^ genome copies (gc) per milliliter in the apical surface at days 1 and 2 in both untreated and treated wells shows that virus entered the tissues. This initial level of virus in the treated wells was expected since peptide was not added until after the entry period of 90 min. In the untreated wells, viral genome copy numbers continued to rise, increasing by 4 log units to the level of 10^8^ gc/ml at day 7. Similarly increasing levels of live virus were detected in these samples. However, in SARS-CoV-2 lipopeptide-treated wells, viral genome copy numbers declined to almost undetectable levels from day 4 to day 7, demonstrating that the initial infection is extinguished in the airway of peptide-treated cells. No infectious virus was detected from the SARS-CoV-2 lipopeptide-treated wells. Interestingly, in the untreated wells, viral genome (10^5^ gc/ml) and infectious virus were detected in the basolateral fluid at day 7, while in the peptide-treated wells, there was no viral exit into the basolateral space.

Viruses from both treated and untreated airway infections at days 3 and 7 were subjected to deep sequencing to identify potential emergence of mutations (see Movie S1 in the supplemental material). No significant mutations were observed in viruses emerging from the peptide-treated airways, and the number of reads in the day 7 treated isolate was nearly as low as in the uninfected negative control, consistent with the low viral load detected by RT-qPCR. The inoculum isolate had minor coding variations at D804G (37% allele frequency) in the nsp12 RNA-dependent RNA polymerase, K339R (4%) in the nsp14 proofreading exonuclease. The untreated day 3 SARS-CoV-2 isolate had slight reductions in allele frequency for the D804G nsp12 variant (26%) and K339R nsp14 variant (28%), as well as three mutations in the S protein: D215G at 32%, P1143S at 22%, and I468V at 15% allele frequencies. The same isolate also contained a nonsense Q418X mutation at a 21% allele frequency in the N gene. The day 7 isolate had a 64% allele frequency H75T mutation in the nsp12 RNA-dependent RNA polymerase.

10.1128/mBio.01935-20.1 MOVIE S1 Supplemental LAVA plot. HTML plot of longitudinal allele frequencies in SARS-CoV-2 genomes from inoculum, day 3 (infection with 20,000 PFU), day 7 untreated (infection with 2,000 PFU) and day 7 (infection with 2,000 PFU) HRC peptide-treated virus. Download Movie S1, HTML file, 3.4 MB. Copyright © 2020 Outlaw et al. 2020 Outlaw et al. This content is distributed under the terms of the Creative Commons Attribution 4.0 International license.

## DISCUSSION

The HRC lipopeptide corresponding to SARS-CoV-2 blocks S-mediated membrane fusion, is effective against both SARS-CoV-2 and MERS-CoV live viruses *in vitro*, blocks spread of SARS-CoV-2 in human airway tissue, and prevents exit of SARS-CoV-2 via the systemic surface (i.e., the basolateral side) of the HAE. Our results contrast with those reported by Xia et al. (39) in that we observe superiority of the native SARS-CoV-2 HRC sequence compared to the designed peptide EK1. It is important to note, however, that the previous comparison involved peptides lacking the cholesterol appendage that we find to be essential for efficacy and involved only cell-cell fusion assays and infectivity assays with pseudotyped virus. The superiority of the native SARS-CoV-2 HRC sequence relative to EK1 in terms of inhibiting SARS-CoV-2 infection may arise because the native complement of hydrophobic residues that pack against the HRN trimer leads to more stable binding of the inhibitor peptide to the transient extended form of the S trimer relative to the hydrophobic residue complement in EK1.

The IC_50_ and IC_90_ values shown in Fig. 2 and 3 suggest that the SARS-CoV-2 HRC lipopeptide is sufficiently potent to enable clinical applications in humans. The efficacy in HAE supports feasibility, since results in this model for other respiratory viruses correlate with efficacy in animals (36, 40).

In the absence of lipopeptide treatment, the presence of virus in the basolateral side of the air-liquid interface in this model suggests that SARS-CoV-2 may exit at this surface and thereby access the systemic circulation. This hypothesis, however, must be subjected to further evaluation (e.g., in engineered tissue platforms that contain a vascular component). Ongoing studies will reveal whether this observation of viral transit to the basolateral airway surface represents events *in vivo*. While the current article was being revised, a study using a similar model was published (41); this report demonstrated basolateral release of the viral progeny as we report here. In this report, side-by-side assessment with another coronavirus (HCoV-NL63) that released particles apically seemed to indicate that this behavior is specific for SARS-CoV-2. The release of particles was associated with partial loss of transepithelial electrical resistance (TEER) (42–45), indicating partial loss of tissue integrity (41). The amount of time postinfection when basolateral release was observed differs between the current work (7 days) and the recent publication (48 h). We also did not observe the significant morphological changes in the infected HAE described in the recent report. We consider the possibility that these differences may result from the different multiplicities of infection (MOI) that were used, ∼0.002 in this work versus 0.1 in the recent report (41).

The availability and density of the receptor for S, angiotensin-converting enzyme 2 (hACE2) (46–48), are presumed to determine viral tropism, along with the availability of plasma membrane-associated type II transmembrane serine protease, TMPRSS2, which is thought to process S after receptor engagement and prior to membrane fusion (49, 50). As we were developing the assays for SARS-CoV-2 S-mediated fusion, we noted that when the fusion assay is conducted with cells that overexpress hACE2, higher concentrations of inhibitory peptide are required to inhibit SARS-CoV-2 S-mediated fusion; when HEK 293T cells that express only baseline levels of hACE2 are used as targets, the IC_50_ for inhibition by the SARS-CoV-2 HRC lipopeptide is ∼0.09 nM (Fig. 2B), rather than ∼10 nM, as observed when the target cells overexpress hACE2. This finding is consistent with our previous studies showing that, for measles virus, higher concentrations of measles virus fusion-inhibitory peptides are required to inhibit fusion in cells overexpressing SLAM or Nectin-4 (receptors for measles receptor-binding protein) (51). Therefore, to provide a stringent model for efficacy and better distinguish between inhibitors, we performed all fusion experiments using receptor-transfected target cells (either hACE2 for SARS-CoV-2 or dipeptidyl peptidase 4 [DDP4] for MERS-CoV). A recent publication reported a lipidated peptide (IPB02) similar in construct to the one described here with inhibition of SARS-CoV-2 S-mediated fusion and infection by pseudotyped virus. IPB02 was derived from residues 1169 to 1203 of SARS-CoV-2 S, one residue shorter at the N terminus than the SARS-CoV-2 HRC peptide described in our work, and a different mode of lipidation, amidation of a C-terminal lysine side chain with cholesteryl succinate, was employed for IPB02 (52). We show here the dramatic difference in potency with and without overexpression of hACE2 receptor. It is unclear whether IPB02 was tested with or without receptor overexpression, making a suitable comparison between the two lipopeptides difficult. The reported inhibitory activity of cell-cell fusion mediated by SARS-CoV-2 S for IPB02 (∼25 nM) is comparable to that of SARS-CoV-2 lipopeptide (∼10 nM) with overexpression of the receptor but significantly less potent than that of the SARS-CoV-2 lipopeptide (∼0.09 nM) without receptor overexpression. Our current study also examines the antiviral efficacy of SARS-CoV-2 lipopeptide on live SARS-CoV-2 virus in both cell monocultures and human airway epithelial tissues. The ability of IPB02 to block infection by live SARS-CoV-2 virus has not yet been reported.

Deep sequencing analysis revealed no significant mutations in viruses emerging from SARS-CoV-2 HRC lipopeptide-treated airway tissues. These data suggest that no resistance mutations emerged under the selective pressure of lipopeptide treatment. Assessing the potential for resistance is important for understanding the feasibility of our proposed prophylactic/therapeutic approach. The clinical use of enfuvirtide for human immunodeficiency virus type 1 (HIV-1) resulted in the emergence of drug-resistant HIV-1 variants (53). Escape variant viruses also emerged upon *in vitro* passaging of HIV-1 in the presence of enfuvirtide (54). The resistant viral population acquired mutations within a highly conserved stretch of three HRN amino acids, glycine-isoleucine-valine (GIV). Resistance mutations in this GIV motif also exist within the viral quasispecies of patients on enfuvirtide therapy. The resistance was due to either decreased interaction between the viral HRN and enfuvirtide or increased interaction between viral HRN and HRC. Attaching a lipid tag to enfuvirtide improved the half-life *in vivo* and partially increased the barrier for resistance (55–61). While anti-SARS-CoV-2 therapy will be of shorter duration than that for HIV (acute versus chronic treatment), resistance may be important clinically, as it is for influenza (62). In the 7 days of the HAE infection, we did not observe significant fixation of mutations in the S protein within lipopeptide-treated airways. In future studies, we will use infections in the HAE model to study the potential for emergence of SARS-CoV-2 viruses resistant to the inhibitory effect of our peptides, as we have done for other viruses (63, 64).

The emergence of SARS-CoV-2 as a global pathogen has created an urgent need for therapeutic agents for prophylaxis and blocking transmission. Entry inhibitors acting at the site of infection are ideal for this purpose. Since our previously developed peptide inhibitors for other respiratory viruses are effective in animals when administered via the intranasal/airway route, with long duration in the lung, this approach is ideal for prophylaxis during exposure and/or to prevent transmission.

The results presented here, taken together with our published data for other viruses (10–12, 15, 23, 40), suggest that an effective prophylactic regimen can be achieved for SARS-CoV-2 and suggest feasibility for treatment after infection as well. *In vivo* experiments in a ferret transmission model will be conducted to determine the potential for the SARS-CoV-2 HRC lipopeptide to prevent transmission. Health care workers and other first responders would benefit directly from our prophylactic approach since it could be easily administered (e.g., once a day intranasally), and based on our experience with other respiratory viruses, protection would be immediate and last for at least 24 h (versus a longer-term vaccine strategy). At-risk individuals (e.g., immunocompromised or with other underlying medical conditions) will also benefit from this prophylaxis. As for HIV, a single antiviral drug is unlikely to be sufficient, and a cocktail of antiviral drugs will likely be the therapeutic path. In addition to being used alone for prophylaxis, our airway-delivered fusion inhibitor peptides could also be used in combination with other drugs as part of a future therapeutic cocktail. Although our initial efforts have focused on combating SARS-CoV-2 infection due to the urgency of the COVID-19 pandemic, the efficacy of the SARS-CoV-2 HRC lipopeptide against both SARS-CoV-1 and MERS suggests promise for the development of a broad-spectrum anticoronavirus agent.

## MATERIALS AND METHODS

### Lipopeptide synthesis.

Peptides corresponding to residues 1168 to 1203 of SARS-CoV-2 S, residues 1251 to 1286 of MERS-CoV S, and EK1 with a C-terminal -GSGSGC spacer sequence were prepared by solid-phase peptide synthesis (SPPS). All peptides were acetylated at the N terminus prior to resin cleavage, followed by purification by reversed-phase high-performance liquic chromatography (HPLC) and characterization by matrix-assisted laser desorption ionization−time of flight mass spectrometry (MALDI-TOF MS). Conjugation to the C-terminal cysteine was effected by the addition of BrAcNH-PEG_4_-Chol to a stirring solution of peptide in dimethyl sulfoxide (DMSO) under nitrogen, followed by *N,N*-diisopropylethylamine (DIEA). The reaction was monitored by ultrahigh-performance liquid chromatography (UPLC) (10 to 95% acetonitrile/H_2_O gradient, 6 min, 50°C). HPLC purification and lyophilization yielded the peptide-lipid conjugates as white powders. Identity was characterized by MALDI-TOF MS.

### Cells.

Human embryonic kidney (HEK) 293T and Vero (African green monkey kidney) cells were grown in Dulbecco’s modified Eagle’s medium (DMEM; Invitrogen; Thermo Fisher Scientific) supplemented with 10% fetal bovine serum (FBS) and antibiotics in 5% CO_2_. Vero E6 cells (ATCC CRL-1586) were grown in minimum essential medium with Earle’s salts (EMEM; Gibco) supplemented with 6% FBS and antibiotics in 5% CO_2_.

### Plasmids.

The cDNAs coding for hACE2 fused to the fluorescent protein Venus, dipeptidyl peptidase 4 (DPP4) fused to the fluorescent protein Venus, SARS-CoV-2 S, SARS-CoV-1 S, and MERS-S (codon optimized for mammalian expression) were cloned in a modified version of the pCAGGS (with puromycin resistance for selection).

### Viruses.

SARS-CoV-2 strain USA_WA1/2020 was obtained from the University of Texas Medical Branch (UTMB) World Reference Center for Emerging Viruses and Arboviruses (WRCEVA) and propagated in Vero E6 cells. Virus stocks were generated from clarified cell culture supernatants harvested 3 or 4 days postinoculation. The recombinant virus expressing neon green (icSARS-CoV-2-mNG) was developed by Pei-Yong Shi and colleagues (38) and propagated in Vero E6 cells. MERS-CoV strain Saudi Arabia 2012 was obtained from Thomas G. Ksiazek (UTMB WRCEVA). All work with infectious virus (propagation, titration, and plaque reduction assays) was done in the biosafety level 3 (BSL3) facility at the Galveston National Laboratory of UTMB.

### β-Gal complementation-based fusion assay.

We previously adapted a fusion assay based on alpha complementation of β-galactosidase (β-Gal) (10). In this assay, hACE2 or DDP4 receptor-bearing cells expressing the omega peptide of β-Gal are mixed with cells coexpressing glycoprotein S and the alpha peptide of β-Gal, and cell fusion leads to alpha-omega complementation. Fusion is stopped by lysing the cells, and after addition of the substrate (Tropix Galacto-Star chemiluminescent reporter assay system; Applied Biosystem), luminescence is quantified on a Tecan M1000PRO microplate reader.

### Viral titration and plaque reduction neutralization assay.

Titers of virus stocks were determined by plaque assay in Vero E6 cells grown in six-well tissue culture plates. Virus stocks were serially diluted 10-fold in PBS, and 0.2 ml of each dilution was inoculated into quadruplicate wells and allowed to adsorb at 37°C for 1 h with rocking every 15 min. Monolayers were rinsed with Dulbecco’s phosphate-buffered saline (DPBS; Corning) and then overlaid with a semisolid medium containing MEM, 5% FBS, antibiotics, and ME agarose (0.6%). Cultures were incubated at 37°C for 3 days and overlaid with DPBS containing neutral red (3.33 g/liter; Thermo Fisher Scientific) as a stain (10%), and plaques were counted after 4 to 5 h.

Peptides were tested for inhibitory activity against SARS-CoV-2 and MERS-CoV by plaque reduction neutralization assay. Peptides were serially diluted in molecular biology grade water (10,000 nM through 5 nM or 1,000 nM through 0.5 nM), each peptide dose was mixed with an equal volume of virus containing 500 particle-forming units (PFU)/ml in MEM, and the peptide/virus mixtures were incubated at 37°C for 1 h. Each peptide dose/virus mixture was inoculated into triplicate wells of Vero E6 cells in six-well plates (0.2 ml per well) and allowed to adsorb at 37°C for 1 h with rocking every 15 min. Monolayers were rinsed with DPBS prior to the addition of medium overlay containing MEM, 5% FBS, antibiotics, and ME agarose (0.6%). Cultures were incubated at 37°C for 3 days and overlaid with medium containing neutral red as a stain, and plaques were counted after 4 to 5 h. Virus controls were mixed with sterile water instead of peptide.

### HAE cultures.

The EpiAirway AIR-100 system (MatTek Corporation) consists of normal human-derived tracheo/bronchial epithelial cells that have been cultured to form a pseudostratified, highly differentiated mucociliary epithelium closely resembling that of epithelial tissue *in vivo*. Upon receipt from the manufacturer, HAE cultures were handled as we have done previously (13, 36, 65). Briefly, cultures were transferred to six-well plates containing 1.0 ml medium per well (basolateral feeding, with the apical surface remaining exposed to air) and acclimated at 37°C in 5% CO_2_ for 24 h prior to experimentation.

### Viral infection of HAE.

HAE cultures were infected by applying 200 μl of EpiAirway phosphate-buffered saline (MatTek TEER buffer) containing 2,000 PFU of infectious-clone-derived SARS-CoV-2 expressing a stable mNeonGreen reporter gene (icSARS-CoV-2-mNG) (38) to the apical surface for 90 min at 37°C. At 90 min, the medium containing the inoculum was removed, the apical surface was washed with 200 μl of TEER buffer, and either 20 μl of peptide (10,000 nM) or an equivalent amount of TEER buffer was added as a treatment. Cultures were fed each day by replenishing 1.0 ml medium on the basolateral side after harvest. The final peptide concentration was 200 nM.

Virus was harvested by adding 200 μl TEER buffer per well to the HAE cultures’ apical surface and allowed to equilibrate for 30 min at 37°C. The suspension was then collected, inactivated with TRIzol reagent (Thermo Fisher) and processed for RT-qPCR. This viral collection was performed sequentially with the same wells of cells on each day postinfection. After harvest of apical and basolateral suspensions, cells were lysed using TRIzol on day 7 postinfection. The amount of infectious virus from HAE supernatants collected from apical and basolateral sides were determined by plaque assay in Vero E6 cells grown in 12-well plates inoculated with 0.1 ml per well (triplicates of each 10-fold dilution in PBS).

### Quantitative RT-PCR.

Viral titers in cell extracts and supernatant fluid were estimated by quantitative RT-PCR (RT-qPCR). Total RNA was extracted using RNeasy minikit according to the manufacturer's instructions (Qiagen). Reverse transcriptions were performed using GoScript reverse transcription system (Promega). Obtained cDNAs were diluted 1:10. Quantitative PCR (qPCR) was performed using Platinum SYBR Green qPCR SuperMix-UDG with ROX kit (Invitrogen). qPCR was run on the ABI 7000 PCR system (Applied Biosystems) using the following protocol: (i) 5 min at 95°C; (ii) 40 cycles with 1 cycle consisting of 15 s at 95°C and 1 min at 60°C; (iii) f a melting curve up to 95°C at 0.8°C intervals. A standard reference (2019-nCoV Positive Control-nCoVPC from “the CDC 2019-nCoV Real-Time” kit) was included in each run to standardize results.

### Cell toxicity assay.

HEK293T or Vero cells were incubated with the indicated concentration of the peptides or vehicle (dimethyl sulfoxide) at 37°C. The cytotoxicity was determined after 24 h using the Vybrant MTT cell proliferation assay kit according to the manufacturer’s guidelines. The absorbance was read at 540 nm using Tecan M1000PRO microplate reader. HAE cultures were incubated at 37°C in the presence or absence of 1, 10, or 100 μM concentrations of the peptide. The peptide was added to the feeding medium. Cell viability was determined on day 7 using the Vybrant MTT cell proliferation assay kit according to the manufacturer’s guidelines. The absorbance was read at 540 using a Tecan M1000PRO microplate reader.

### RNA-seq.

Total RNA from SARS-CoV-2-infected HAE cells was purified using Direct-zol RNA MicroPrep kit. RNA concentration and RNA integrity number (RIN) were determined using an Agilent microfluidic RNA 6000 Nano Chip kit (Agilent Technologies, Santa Clara, CA) on a 2100 bioanalyzer (Agilent Technologies, Santa Clara, CA). Those samples with a RIN of greater than 9 were used for transcriptome sequencing (RNA-seq). Poly(A) pulldown was used to enrich mRNAs from total RNA samples. Libraries were prepared using Illumina TruSeq RNA prep kit (Illumina, San Diego, CA). Libraries were then sequenced using the Illumina HiSeq2000 instrument (Illumina, San Diego, CA) at the Columbia Genome Center. Inoculum RNA was sequenced with total RNA shotgun sequencing as described previously (66). Coding variants were called using bwa-mem (see https://arxiv.org/abs/1303.3997) and LAVA (67) using NC_045512.2 as the reference genome and the inoculum as the initial isolate. Variants detected by LAVA, as well as additional variants in ORF1ab were manually confirmed using Geneious 11.1.5 (68).

### Data availability.

SARS-CoV-2 sequencing reads are available under NCBI BioProject accession number PRJNA634194.
