# Supplementary figures and images for: Inhibition of Coronavirus Entry In Vitro and Ex Vivo by a Lipid-Conjugated Peptide Derived from the SARS-CoV-2 Spike Glycoprotein HRC Domain
Source: mBio. 2020 Oct 20;11(5):e01935-20. doi: 10.1128/mBio.01935-20 (PMC7587434; doi:10.1128/mBio.01935-20)

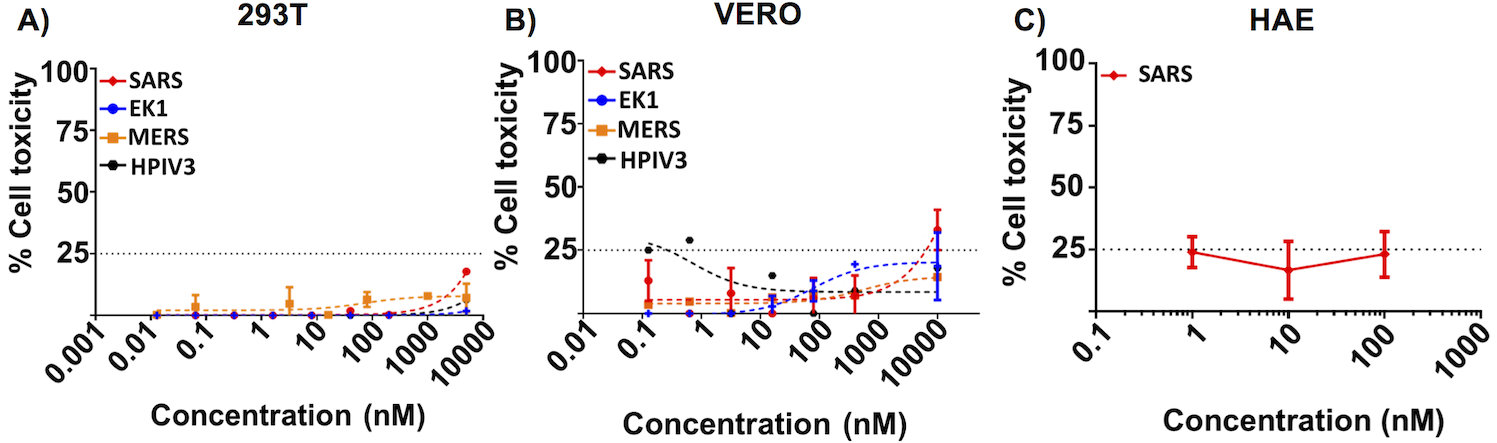

Supplement: FIG S1 [file mBio.01935-20-sf001.tif]
