# Supplementary material for: Inhibition of Coronavirus Entry In Vitro and Ex Vivo by a Lipid-Conjugated Peptide Derived from the SARS-CoV-2 Spike Glycoprotein HRC Domain
Source: mBio. 2020 Oct 20;11(5):e01935-20. doi: 10.1128/mBio.01935-20 (PMC7587434; doi:10.1128/mBio.01935-20)
Supplement: MOVIE S1 [file mbio01935-20_Supp_1_seq8.html.zip › mbio01935-20_Supp_1_seq8.html]

SARS-CoV-2 HRC
